# Supplementary material for: Combined Soluble Fiber-Mediated Intestinal Microbiota Improve Insulin Sensitivity of Obese Mice
Source: Nutrients. 2020 Jan 29;12(2):351. doi: 10.3390/nu12020351 (PMC7071167; doi:10.3390/nu12020351)
Supplement: Supplementary file 1 [file nutrients-12-00351-s001.pdf]

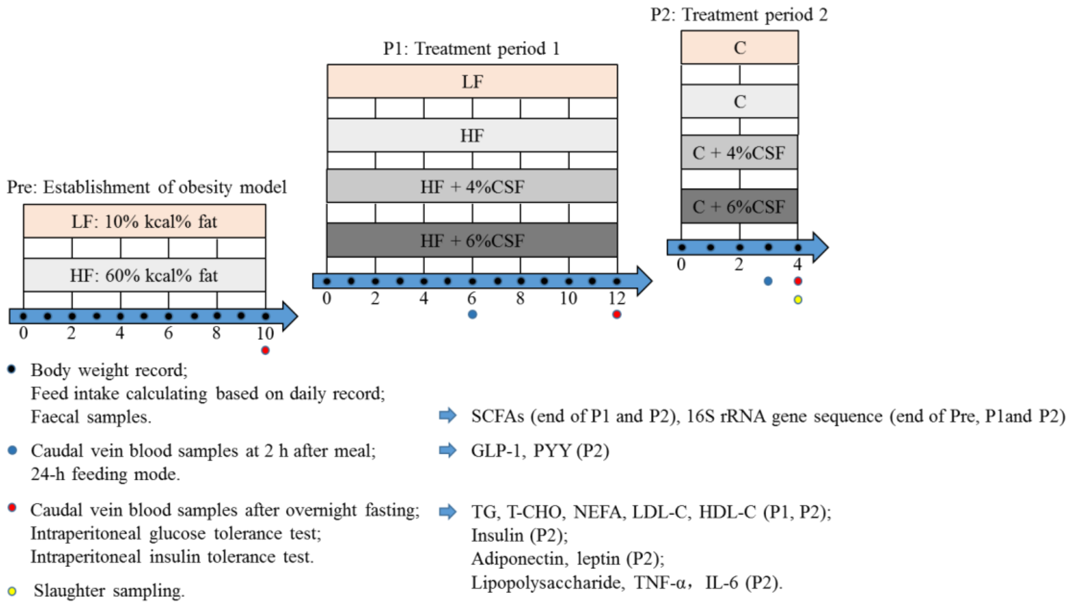

Figure S1. Overview of the study design and sample collection.

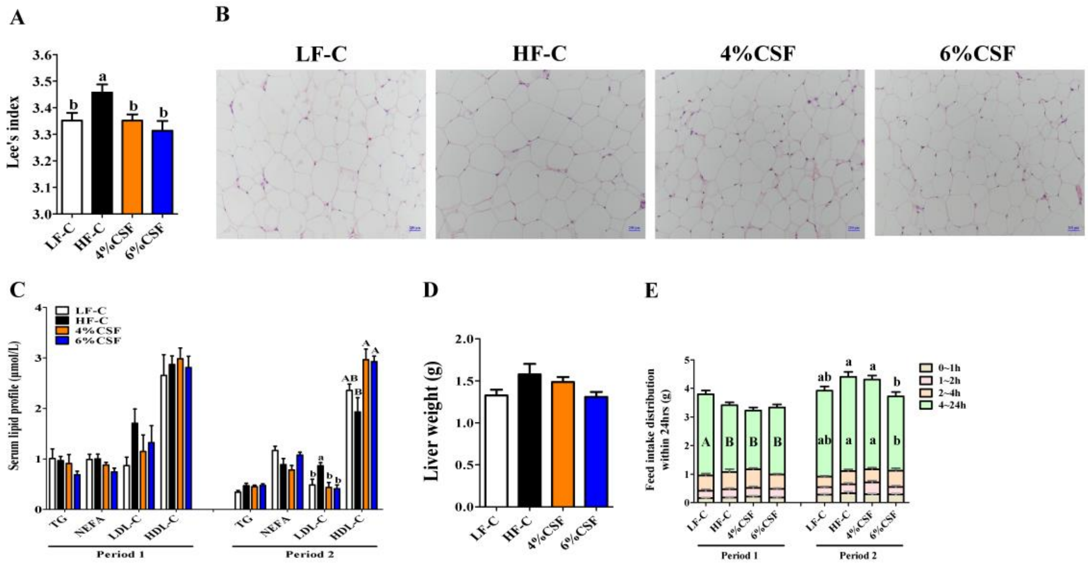

Figure S2, related to figure 1. Effects of CSF on adiposity and appetite of DIO mice within a two-stage feeding scheme. DIO Mice were fed HFD, or HFD supplemented with 4% or 6%CSF for 12 weeks within period 1, and then fed chow diet (CD) or CD supplemented with 4% or 6%CSF for 4 weeks within period 2 (n = 7/group). Normal mice (n = 7) were fed LFD during period 1 and CD during period 2. (A) Lee's index at termination of study. Lee's index was calculated using third power of body weight (g) divided by nasoanal length (cm). (B) Representative H&E stained images of epididymal WAT at 200× magnification. Scale bars, 200 μm. (C) Serum lipid profile. (D) Liver weight. (E) 24-hour feeding pattern. Data are expressed as mean ± SEM. Statistical significance was assessed by Tukey's test for multiple comparisons. ab means in the same bar without a common letter differ at $P < 0.05$; AB means in the same bar without a common letter differ at $P < 0.01$.

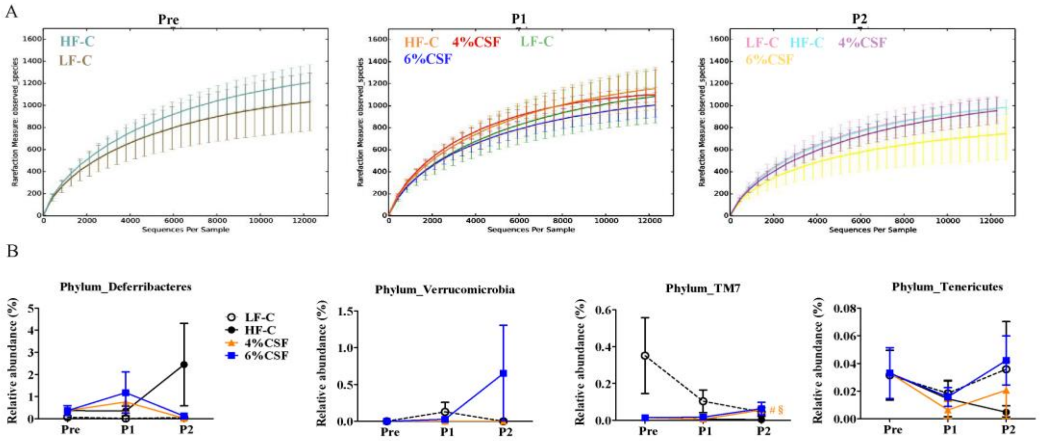

Figure S3, related to figure 4. CSF alters the gut microbiota composition. (A) The rarefaction curves of observed species of faeces microbiota over time. (B) The changes of relative abundance of Deferribacteres, Verrucomicrobia, TM7 and Tenericutes. Data are expressed as mean ± SEM. Statistical significance was assessed by unpaired Student t test. &,&& P < 0.05, 0.01, respectively, compared with the LF-C group. §, P < 0.05, compared with the treatment period 1. The orange and blue statistical symbols represent the statistical analysis of 4% CSF or 6% CSF groups, respectively, with other groups or the same group at different time points.

Table S1. The composition of the purified diets used in this study

Experimental stage	Treatment period 1				Treatment period 2			
Group	LF-C	HF-C	4%CSF	6%CSF	LF-C	HF-C	4%CSF	6%CSF
Basal diet	TP23302	TP23300			LAD3001M			
	low fat	high fat diet			chow diet			
kcal/gm	3.60	5.00			3.60			
Protein, kcal%	19.00	19.40			14.10			
Carbohydrate, kcal%	71.00	20.60			75.90			
Fat, kcal%	10.00	60.00			10.00			
**Ingredient**								
Casein, gm	191.00	267.00	267.00	267.00	140.00	140.00	140.00	140.00
Corn starch, gm	497.00	0.00	0.00	0.00	465.69	465.69	465.69	465.69
Maltodextrin, gm	112.00	157.00	157.00	157.00	145.00	145.00	145.00	145.00
Sucrose, gm	64.00	89.00	89.00	89.00	100.00	100.00	100.00	100.00
Fat, gm	40.00	334.00	334.00	334.00	40.00	40.00	40.00	40.00
Cellulose, gm	48.00	67.00	27.00	7.00	60.00	60.00	20.00	0.00
CSF, gm	0.00	0.00	40.00	60.00	0.00	0.00	40.00	60.00
Vitamin Mix,V1010 and	43.00	79.00	79.00	79.00	45.00	45.00	45.00	45.00
L-Cystine, gm	3.00	4.00	4.00	4.00	1.80	1.80	1.80	1.80
Choline Bitartrate, gm	2.00	3.00	3.00	3.00	2.50	2.50	2.50	2.50
TBHQ, gm	0.01	0.07	0.07	0.07	0.01	0.01	0.01	0.01
Total, gm	1000.01	1000.07	1000.07	1000.07	1000.00	1000.00	1000.00	1000.00
**Laboratory analysis**								
Gross energy, kcal/gm	4.23	5.87	5.83	5.81	4.39	4.39	4.27	4.21

27 **Table S2.** Key Resource Table-supplemental oligonucleotides.

OLIGONUCLEOTIDES	SOURCE	Pubmed ID
36B4: 5′-TCCAGGCTTTGGGCATCA-3′ and 5′-CTTTATTCAGCTGCACATCACTCAGA-3′	Wuhan AuGCT DNA-SYN Biotechnology Co., Ltd	XR_002380971.2
ZO-1: 5′-ACCCGAAACTGATGCTGTGGATAG-3′ and 5′-AAATGGCCGGGCAGAACTTGTGTA-3′	Wuhan AuGCT DNA-SYN Biotechnology Co., Ltd	BC138028.1
ZO-2: 5′-GTTTGGCCCCATAGCAGATA-3′ and 5′-TCCGGGTTGAAGAAAATCAC-3′	Wuhan AuGCT DNA-SYN Biotechnology Co., Ltd	AF113005.1
Occludin: 5′-ATGTCCGGCCGATGCTCTC-3′ and 5′-TTTGGCTGCTCTTGGGTCTGTAT-3′	Wuhan AuGCT DNA-SYN Biotechnology Co., Ltd	U49185.1
Claudin-1: 5′-ATTTACTCCTATGCCGGCGA-3′ and 5′-ACCTCATCGTCTTCCAAGCA-3′	Wuhan AuGCT DNA-SYN Biotechnology Co., Ltd	NM_021101.5
Claudin-2: 5′-GTCATCGCCCATCAGAAGAT-3′ and 5′-ACTGTTGGACAGGGAACCAG-3′	Wuhan AuGCT DNA-SYN Biotechnology Co., Ltd	NM_016675.4
Claudin-3: 5′-GAGATGGGAGCTGGGTTGTA-3′ and 5′-ACGTAGTCCTTGCGGTCGTA-3′	Wuhan AuGCT DNA-SYN Biotechnology Co., Ltd	NM_009902.4
Claudin-15: 5′-GCCTCTTTCTAGGCATGGTG-3′ and 5′-TCCAGCATACAGTGGGTTGA-3′	Wuhan AuGCT DNA-SYN Biotechnology Co., Ltd	NM_021719.4
Muc-1: 5′-CTGTTCACCACCACCATGAC-3′ and 5′-CTTGGAAGGGCAAGAAAACC-3′	Wuhan AuGCT DNA-SYN Biotechnology Co., Ltd	NM_013605.2
Muc-2: 5′-CAACAAGCTTCACCACAATCTC-3′ and 5′-CAGACCAAAAGCAGCAAGGTA-3′	Wuhan AuGCT DNA-SYN Biotechnology Co., Ltd	NM_023566.3
E-cadherin: 5′-ATCCTCGCCCTGCTGATT-3′ and 5′-ACCACCGTTCTCCTCCGTA-3′	Wuhan AuGCT DNA-SYN Biotechnology Co., Ltd	BC098501.1
16S rRNA: 5′-AGAGTTTGATCCTGGCTCAG-3′ and 5′-CTGCTGCCTCCCGTAGGAGT-3′	Wuhan AuGCT DNA-SYN Biotechnology Co., Ltd	AP019773.1

28

Table S3. Key Resource Table-critical commercial assays.

Critical Commercial Assays		
Triglyceride assay kit	Nanjing Jiancheng Bioengineering Institute, Nanjing, China	Cat.# A110-1-1
Nonesterified Free fatty acids assay kit	Nanjing Jiancheng Bioengineering Institute, Nanjing, China	Cat.# A042-2-1
Low-density lipoprotein cholesterol assay kit	Nanjing Jiancheng Bioengineering Institute, Nanjing, China	Cat.# A113-1-1
High-density lipoprotein cholesterol assay kit	Nanjing Jiancheng Bioengineering Institute, Nanjing, China	Cat.# A112-1-1
Mouse Insulin(INS) ELISA Kit	Jiyinmei Biotechnology Co. Ltd., Wuhan, China	Cat.# JYM0351MO
Mouse Peptide YY(PYY) ELISA Kit	Jiyinmei Biotechnology Co. Ltd., Wuhan, China	Cat.# JYM0764MO
Mouse Glucagon-like peptide 1 (GLP-1) ELISA Kit	Jiyinmei Biotechnology Co. Ltd., Wuhan, China	Cat.# JYM0515MO
Mouse Lipopolysaccharide (LPS) ELISA Kit	Jiyinmei Biotechnology co., Ltd., Wuhan, China	Cat.# JYM0588MO
Mouse Leptin(LEP) ELISA Kit	Jiyinmei Biotechnology co., Ltd., Wuhan, China	Cat.# JYM0015MO
Mouse Adiponectin(ADP) ELISA Kit	Jiyinmei Biotechnology co., Ltd., Wuhan, China	Cat.# JYM0454MO
MU30030 MouseTumor necrosis factor α(TNF-α) ELISA Kit	Bioswamp Biotechnology co., Ltd., Wuhan, China	Cat.# MU30030
Mouse Interleukin(IL-6) ELISA Kit	Bioswamp Biotechnology co., Ltd., Wuhan, China	Cat.# MU30044
BrdU	Servicebio, Wuhan, China	Cat.# WB8010
organic acids	Fluka, Buchs, Switzerland	-
QIAamp Fast DNA Stool Mini Kit	Qiagen, Hilden, Germany	Cat.# 51604
QuantiFast SYBR Green PCR kit	Qiagen, Hilden, Germany	Cat.# 204054
Hieff qPCR SYBR Green Master Mix	Yeasen Biotechnology Co., Ltd., Shanghai, China	Cat.# 11202ES08
TRIzol	Invitrogen, Carlsbad, CA, USA	-
